# Supplementary material for: Attrition from HIV treatment after enrollment in a differentiated service delivery model: A cohort analysis of routine care in Zambia
Source: PLoS One. 2023 Mar 14;18(3):e0280748. doi: 10.1371/journal.pone.0280748 (PMC10013882; doi:10.1371/journal.pone.0280748)
Supplement: S1 Fig — (DOCX) [file pone.0280748.s001.docx]

**S1 Fig. Cumulative incidence curves by stratified groups**

We used a competing risk regression analysis and the risk of attrition was calculated by accounting transferred out as a competing risk of attrition. Below are the cumulative incidence curves of attrition and transferred out over time (months) by stratified groups; most DSD models show a similar or greater proportion of attrition than the proportion of transferred out while the extended clinic hours model (and community pick-up points in some subgroup) show a greater proportion of attrition than the proportion of transferred out. These resulted in a significant reduction of relative risk of attrition compared to the reference group in the regression analysis. (Figure 2 and 3)

1. **≤2 months dispensing**

**
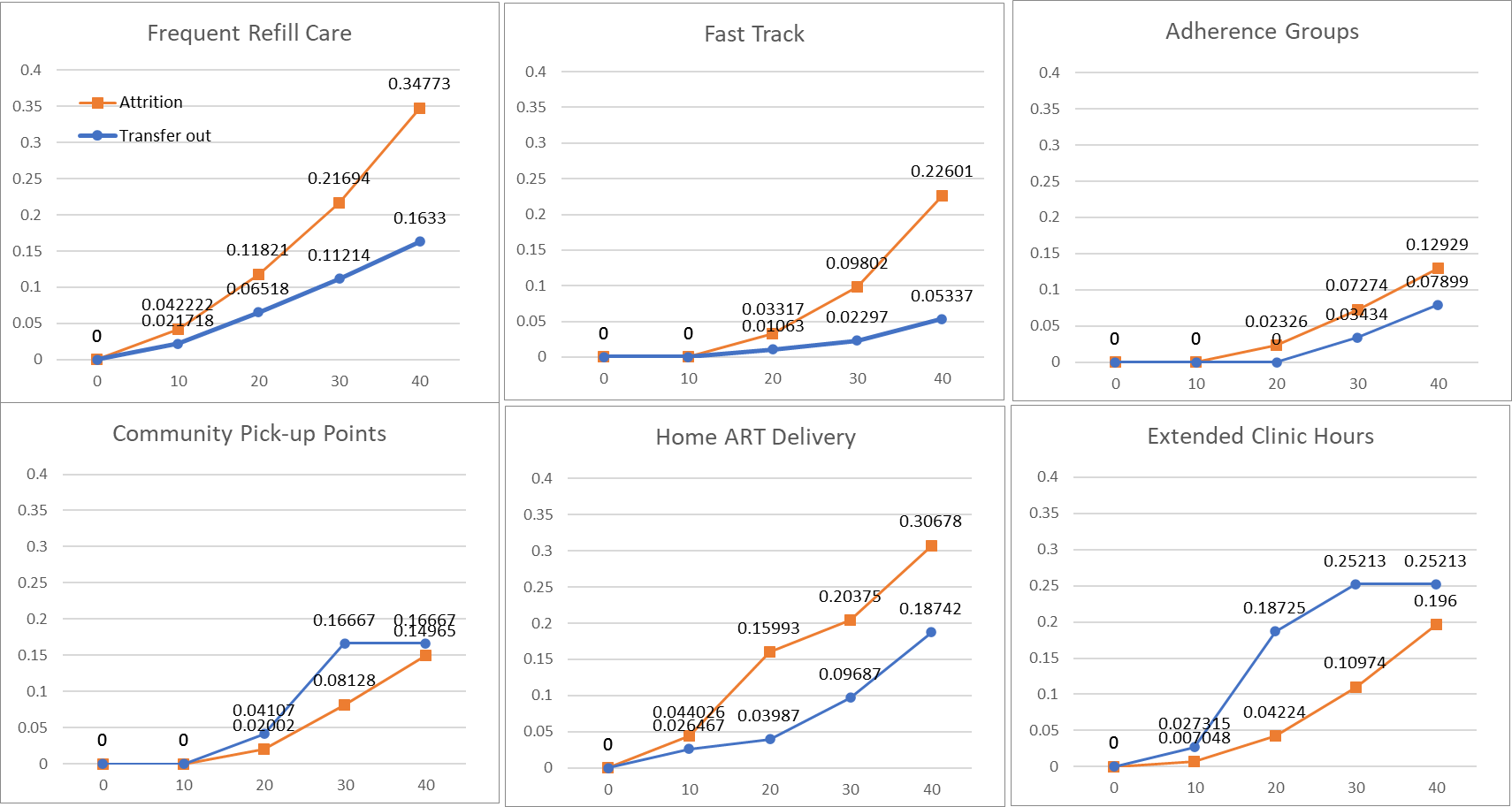
**

1. **3 months dispensing**

**
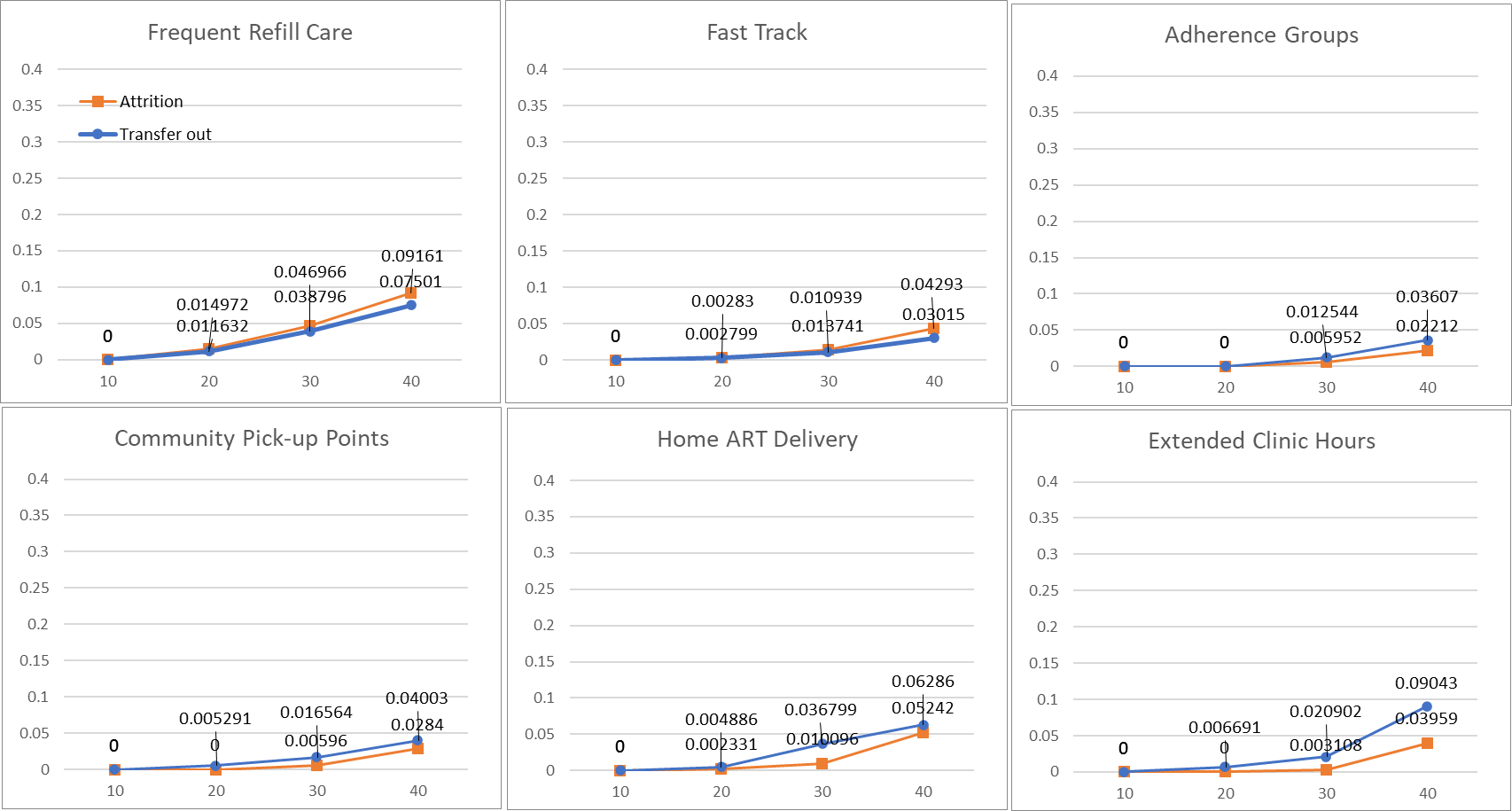
**

1. **4-6 months dispensing**

**
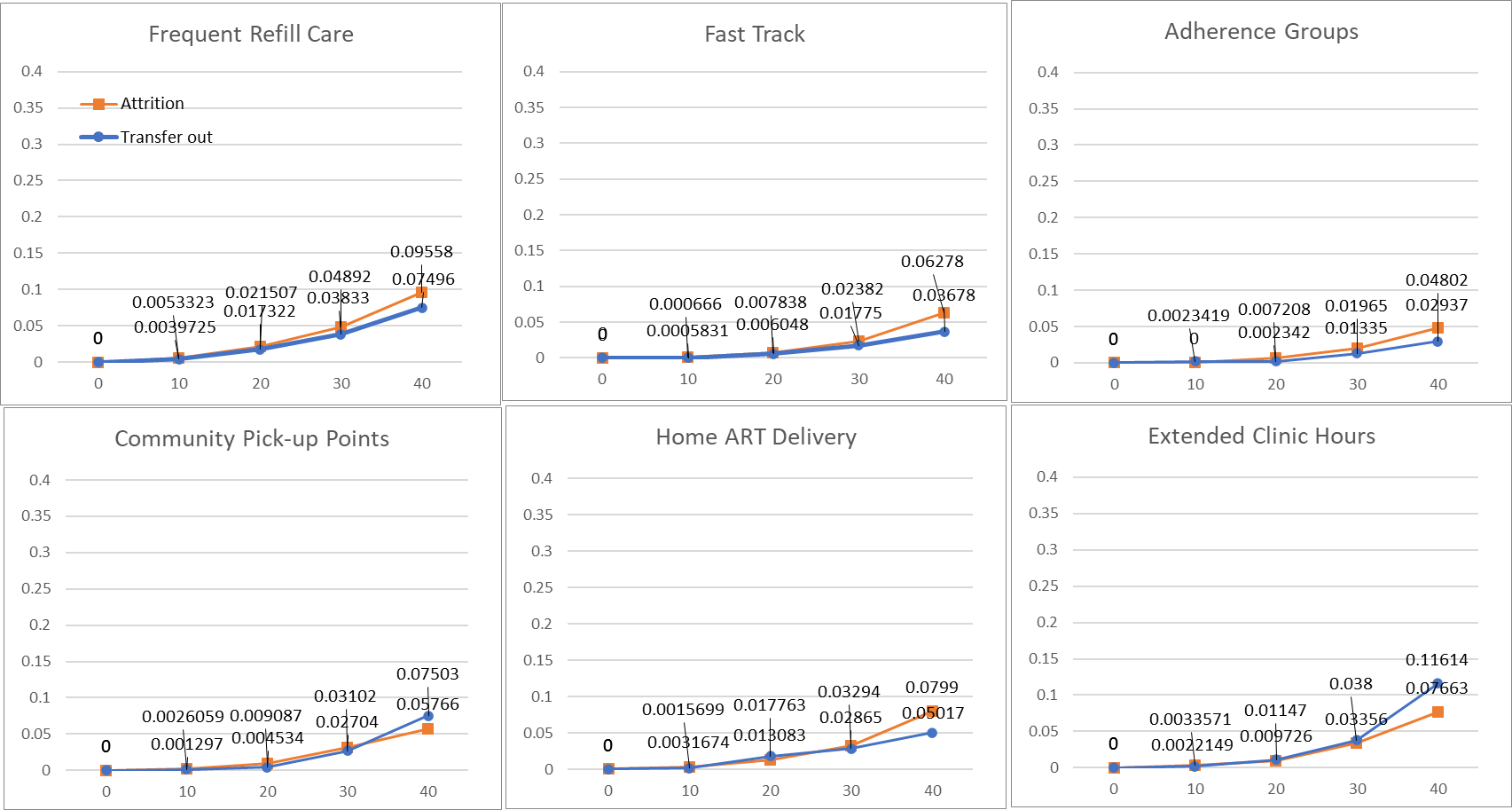
**

1. **Urban female**

**
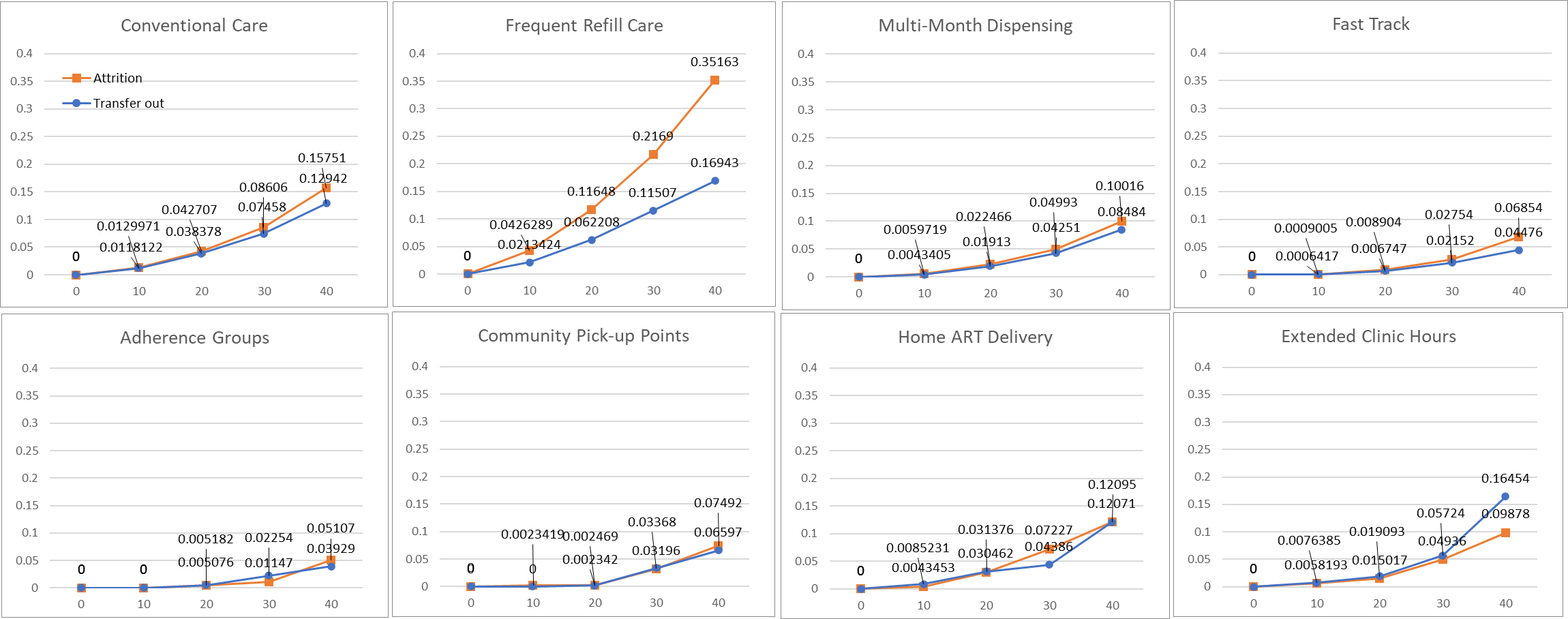
**

1. **Urban male**

**
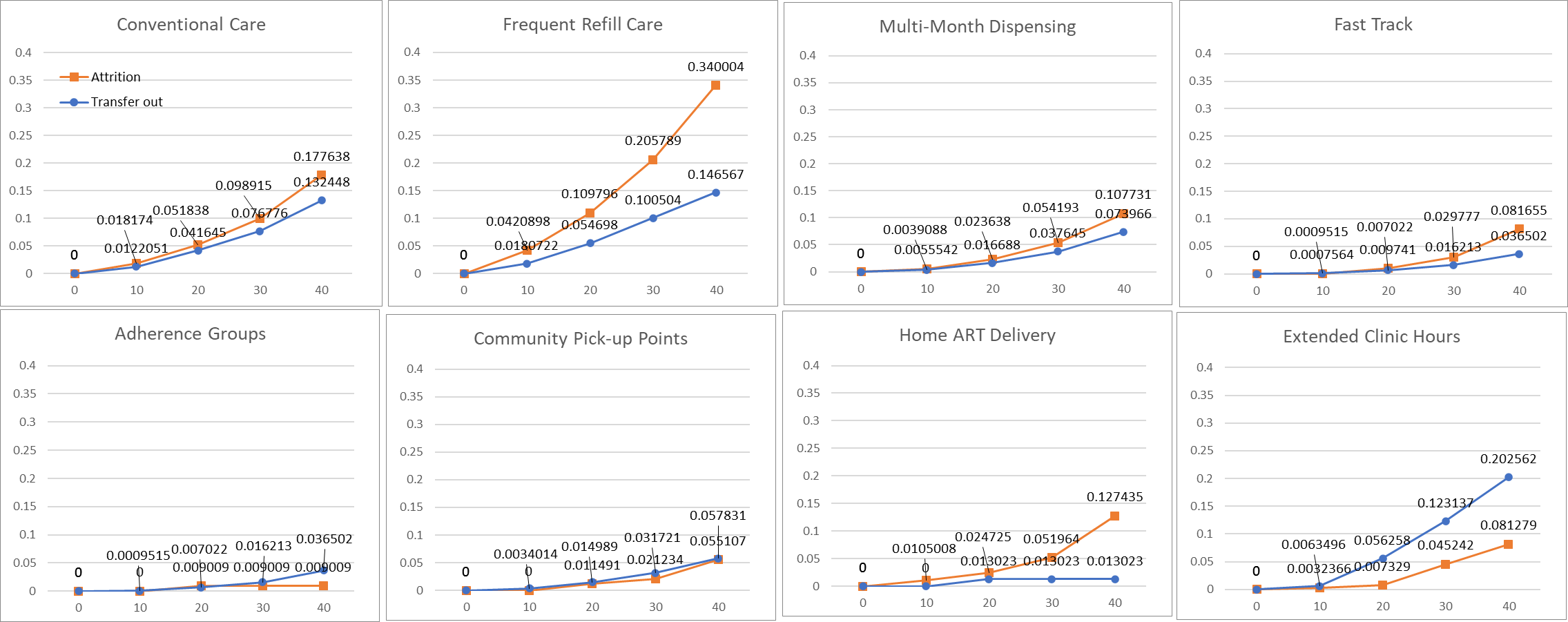
**

1. **Rural female**

**
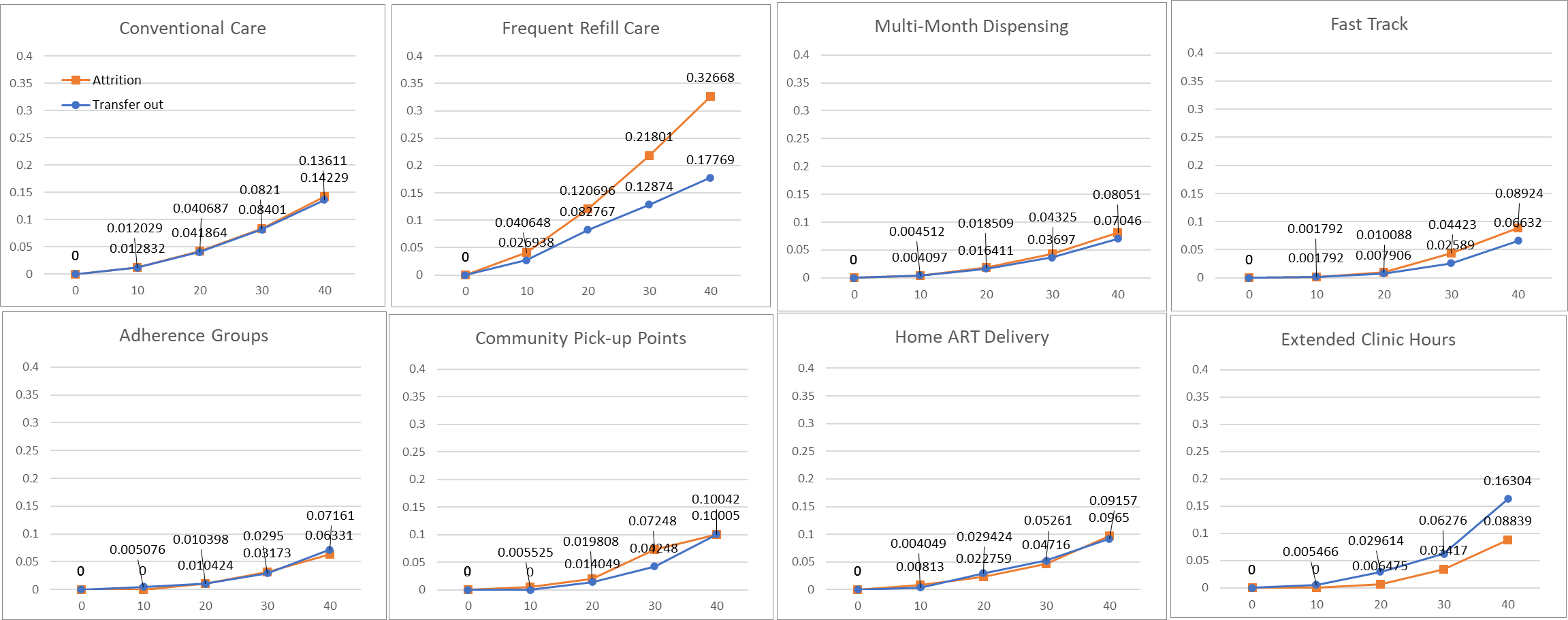
**

1. **Rural male**

**
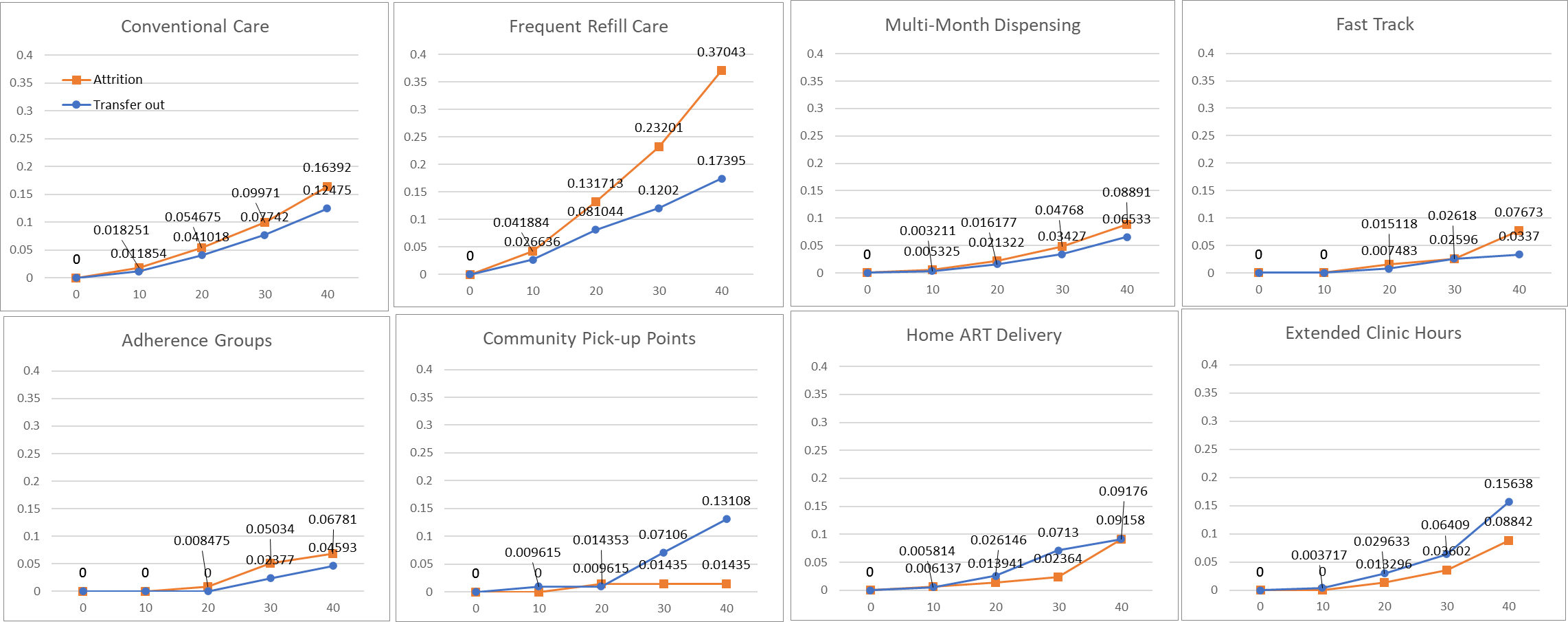
**
